# S149R, a novel mutation in the *ABCD1* gene causing X-linked adrenoleukodystrophy

**DOI:** 10.18632/oncotarget.20974

**Published:** 2017-09-18

**Authors:** Fang Yan, Wenbo Wang, Hui Ying, Hongyu Li, Jing Chen, Chao Xu

**Affiliations:** ^1^ Department of Pain Management, Shandong Provincial Hospital Affiliated to Shandong University, Jinan, Shandong 250021, China; ^2^ Department of Endocrinology and Metabolism, Shandong Provincial Hospital Affiliated to Shandong University, Jinan, Shandong 250021, China; ^3^ Institute of Endocrinology, Shandong Academy of Clinical Medicine, Jinan, Shandong 250021, China; ^4^ Shandong Clinical Medical Center of Endocrinology and Metabolism, Jinan, Shandong 250021, China; ^5^ Department of Pain Management, Ankang City People's Hospital, Ankang, Shanxi 725000, China; ^6^ Department of Child Health, Xiamen Maternal and Child Health Hospital, Xiamen, Fujian 361003, China

**Keywords:** X-linked adrenoleukodystrophy, ABCD1 gene, peroxisomal disorder, genetic diagnosis, bioinformatics analysis

## Abstract

X-linked adrenoleukodystrophy (X-ALD) is the most common peroxisomal disorder. It is a heterogeneous disorder caused by mutations in the *ATP-binding cassette protein subfamily D1* (*ABCD1*) gene, encoding the peroxisomal membrane protein ALDP, which is involved in the transmembrane transport of very long-chain fatty acids. For the first time, we report a case of olivopontocerebellar X-ALD on the Chinese mainland. In this study, a novel mutation (c.447T>A; p.S149R) in *ABCD1* was detected in a patient diagnosed with X-ALD. The mutant amino acid is well conserved among species. Bioinformatics analysis predicted the substitution to be deleterious and to cause structural changes in the adrenoleukodystrophy protein. Immunofluorescence showed an altered subcellular localization of the S149R mutant protein, which may lead to defects in the degradation of very long chain fatty acids in peroxisomes. We therefore suggest that the novel mutation, which alters ALDP structure, subcellular distribution and function, is responsible for X-ALD.

## INTRODUCTION

X-linked adrenoleukodystrophy (X-ALD; MIM: #300100) is the most common inherited peroxisomal disorder characterized by neurodegeneration and adrenal insufficiency. Due to the X-linked recessive inheritance pattern, the majority of patients are males. Remarkably, the minimum incidence of X-ALD among males is reported to be 1:17000 with no apparent difference between countries. However, the incidence in China is not available [1]. X-ALD is classified based on phenotypic expression and the onset of initial symptoms [2, 3]. In male patients, X-ALD is classified as cerebral ALD (childhood, adolescent and adult forms), adrenomyeloneuropathy (AMN), Addison-only (AO), olivopontocerebellar ALD and asymptomatic ALD. In heterozygous females, it is categorized into mild myelopathy, moderate to severe myeloneuropathy, cerebral involvement, clinically evident adrenal insufficiency and asymptomatic ALD [4]. Except for cerebral ALD and AMN, olivopontocerebellar ALD is an exceedingly rare form (1-2% of ALD cases), which is characterized by cerebellar and brainstem involvement in adolescence or adulthood [4]. Symptoms include cerebellar ataxia, gait disturbance and speech disturbance.

X-ALD is related to mutations in the ATP-binding cassette protein subfamily D1 (*ABCD1*: NM_000033.3) gene, which is located on Xq28 and contains 10 exons [5]. The gene encodes the adrenoleukodystrophy protein (ALDP: NP_000024), a transporter in the peroxisome membrane [6]. ALDP contains 745 amino acids, six transmembrane regions and an ATP-binding region, and it is responsible for transporting very long chain fatty acid (VLCFA) CoA esters into the peroxisome [7, 8]. Thus, the dysfunction of ALDP will affect the degradation of VLCFAs in the peroxisome and lead to an increasing concentration of VLCFAs, which will likely cause lesions by inflammatory reactions and oxidative stress [9, 10]. VLCFAs mainly accumulate in myelin, the spinal cord, peripheral nerves, adrenal cortex and testis. Thus, the clinical manifestations include demyelinating disease, adrenocortical insufficiency (Addison's disease) and hypogonadism [7, 11, 12]. Presently, there are approximately 1794 mutations in the *ABCD1* gene, which can be found in the database (http://www.x-ald.nl). Among those reported mutations, 61% are missense, 22% are frameshift, 10% are nonsense and the remaining 7% are insertions or deletions. Mutation analysis is the gold standard for diagnosis, and the genotype does not show necessary relationships to the phenotype. Therefore, further study on the relationship between mutations and clinical features is significant for counseling [13, 14].

In this study, we reported a patient with olivopontocerebellar ALD, which is the first case on the Chinese mainland. Moreover, a novel mutation was identified. Specifically, a bioinformatics analysis and functional study was performed and showed that the mutation destroyed protein function. Our findings will assist in our understanding of the disease and benefit prenatal diagnosis and clinician work.

## RESULTS

### Patient characteristics

The patient was a 30-year-old man who was referred to our hospital (Shandong Provincial Hospital Affiliated to Shandong University, China) for the treatment of ataxia. Six months before admission, he showed weakness in the left lower limb and stumbled. One month later, both legs were weak and he could not stand by himself. Three months before admission, he developed dysarthria, dysphagia and involuntary head shaking. He was born without complications to a nonconsanguineous family, and his family history included no neurological disease. Hyperpigmentation was noticed on his palms and prothorax. Neurological examination in the hospital showed decreased light reflex, restricted abduction of the eyes and nystagmus. Pathological reflexes, including Babinski, Chaddock and Hoffman of both sides, were observed. Motor power was graded as four of five at the lower extremities and five of five at the upper extremities. Muscle tone was high. Tendo jerk of the left upper limb was ++++ and others were +++. Romberg's sign was apparent. The patient could not complete the both hands alternating movement test, the heel-knee-tibia test, or the finger-nose test, which suggested ataxia. The patient was subjected to examinations, including laboratory tests, brain magnetic resonance imaging (MRI) and abdominal computed tomography (CT).

### Clinical features and diagnosis

The laboratory results are summarized in Table 1. The level of ACTH is more than three times than normal level, but cortisol was within the normal range. Testosterone decreased to half of the normal range, and low-density lipoprotein was slightly higher than the normal level.

**Table 1:** Laboratorial evaluation at diagnosis

Test		Result	Change	Reference range
Sex hormone	ACTH(pg/ml)	211	↑	7.2-63.3
	Cortisol 8a.m.(nmol/L)	470.9	N	171-536
	FSH(mIU/ml)	17.34	N	
	LH(mIU/ml)	34.1	N	
	E2(pg/ml)	<5.00	N	
	P(ng/ml)	0.28	N	
	Testosterone(ng/ml)	1.36	↓	2.8-8.0
	PRL(ng/ml)	9.11	N	4.79-23.30
	Dehydroepiandrosterone sulfate (umol/L)	1.35	↓	4.34-12.2
	SHBG(nmol/L)	174.8	↑	14.5-48.4
	FTI(%)	2.7	↓	35.0-92.6
Lipids	LDL(mmol/L)	3.52	↑	0.5-3.36
	HDL(mmol/L)	1.06	N	0.8-1.5
	TC(mmol/L)	5.23	N	3.6-6.2
	TG(mmol/L)	1.28	N	0.4-1.8
	ApoA(g/L)	1.12	N	1.0-2.0
	ApoB(g/L)	0.94	N	0.6-1.1
	ApoA/B	1.19	N	1.0-2.0
	Lpa(g/L)	0.256	N	0-0.3
	FFA(mmol/L)	0.66	N	0.1-0.9

ACTH: adrenocorticotropic hormone; FSH: follicle-stimulating hormone; LH: luteinizing hormone; E2: estradiol; P: progestogen; PRL: prolactin; SHBG: sex hormone binding globulin; FTI: free testosterone index; LDL: low density lipoprotein; HDL: high density lipoprotein; TG: triglyceride; ApoA: apolipoprotein A; ApoB: apolipoprotein B; Lpa: lipoprotein a; FFA: free fatty acid; “↓”: decreased; “↑”: increased; N: normal range.

**Figure 1:**
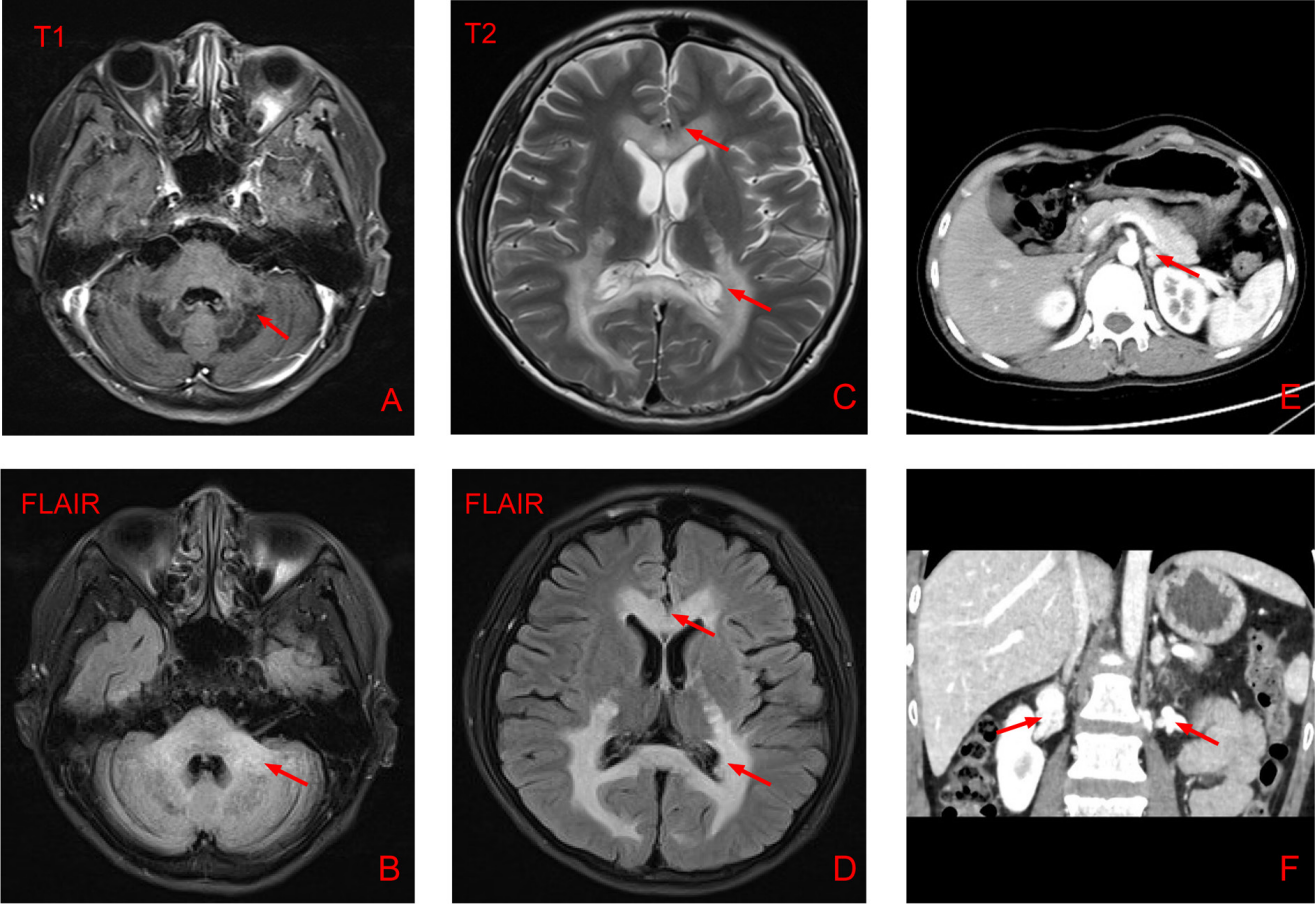
Results of brain magnetic resonance imaging and abdominal computed tomography **(A-D)** Brain MRI of the patient showing multiple patchy symmetrical subtle T2-weighted high-signal-intensity, T1-weighted low-signal-intensity and T2-FLAIR-high-signal-intensity lesions in the bilateral white matter around the lateral ventricle, posterior limbs of the internal capsule, and genu and splenium of the corpus callosum, brainstem and cerebellum. Mild enlargement of the bilateral third ventricle, fourth ventricle and lateral ventricle was observed. **(E-F)** Abdominal CT showing nodular hyperplasia in the bilateral adrenal glands and calcification in the left gland.

The brain MRI reported multiple patchy symmetrical subtle T2-weighted high-signal-intensity and T1-weighted low-signal-intensity lesions in the bilateral white matter around the lateral ventricle, posterior limbs of the internal capsule, and genu and splenium of the corpus callosum, brainstem and cerebellum. T2-FLAIR revealed high signals, DWI reported no apparent restricted diffusion, and SWI showed no increase in signals. No apparent enhancement was observed after GD-DTPA injection. Mild enlargement of the bilateral third ventricle, fourth ventricle and lateral ventricle was observed. No apparent broadening or deepening of the sulcus and cisterns were reported. The middle line structure did not shift. Abdominal CT revealed nodular hyperplasia in bilateral adrenal glands and calcification in the left gland (Figure 1).

The diagnosis was based on clinical, serological and radiological parameters. We suspected olivopontocerebellar ALD because the patient showed adrenal insufficiency (hyperpigmentation and increased ACTH), hypogonadism (low testosterone level) and special neurological manifestations (neurological test and cerebral MRI).

### Treatment

During hospitalization, he received neurotrophic drugs and hormone replacement therapy, including Mecobalamine tablets (1.5 mg/d), Vitamin B1 tablets (30 mg/d), Hydrocortisone tablets (20 mg at 8 a.m., 10 mg at 4 p.m.) and Omeprazole (10 mg/d). He was also asked to have a low-VLCFA diet. After the treatment, the symptoms were slightly improved and he requested discharge from the hospital. Six months after discharge, the symptoms were not relieved. In fact, no effective therapy has been reported for this rare form of ALD. Nevertheless, early diagnosis will benefit genetic counseling and proper management [15, 16].

### Mutation analysis

**Figure 2:**
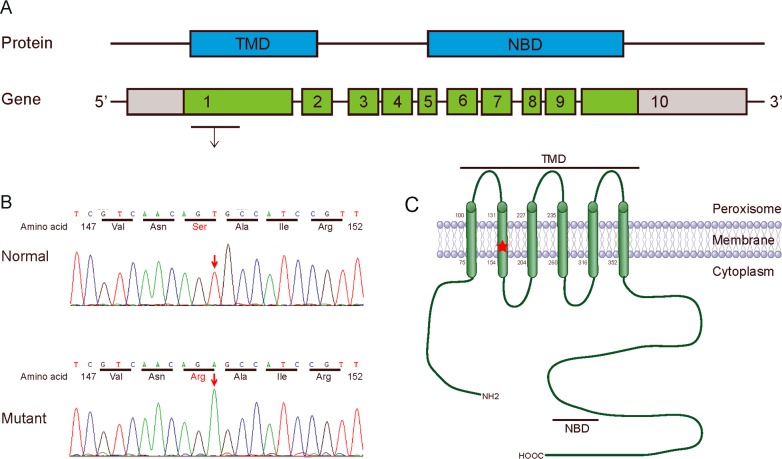
Mutation analysis of *ABCD1* gene **(A)** Protein (ALDP) and gene (*ABCD1*): Genomic structure of the human *ABCD1* gene. The gene consists of 10 exons (boxes) with the start codon in exon 1. Red letters in the gene indicate the position of the missense mutation, which is located in exon 1 of the *ABCD1* gene. **(B)** Sequencing diagram of part of exon 1 in the *ABCD1* gene. The arrow indicates the involved nucleotide change. The upper panel is the normal sequence, whereas the lower panel is the mutated sequence. **(C)** Schematic of the ALDP molecular structure. ALDP contains a TMD (amino acids 75-352) and a nucleotide-binding domain (NBD, also named the ATP-binding region, amino acids 474-700). The red X mark shows the position of the mutation S149R in exon 1 of the *ABCD1* gene.

We revealed a novel nucleotide substitution, T→A, at nucleotide 447 in exon 1 of the *ABCD1* gene, which led to a predicted amino acid residue change from Ser to Arg at codon 149 in this patient in a hemizygote state (Figure 2A and 2B). No other mutations were detected. However, we failed to contact the other members of his family. We ruled out the possibility of polymorphism at this position by screening 100 controls. We also searched dbSNP, ESP6500, HGMD and 1000 genome and found no record of this mutation. As the mutation is located in the second transmembrane domain (TMD2) of ALDP (Figure 2C), a region critical for protein structure and function, we performed the following bioinformatics analysis.

### Bioinformatics analysis

**Figure 3:**
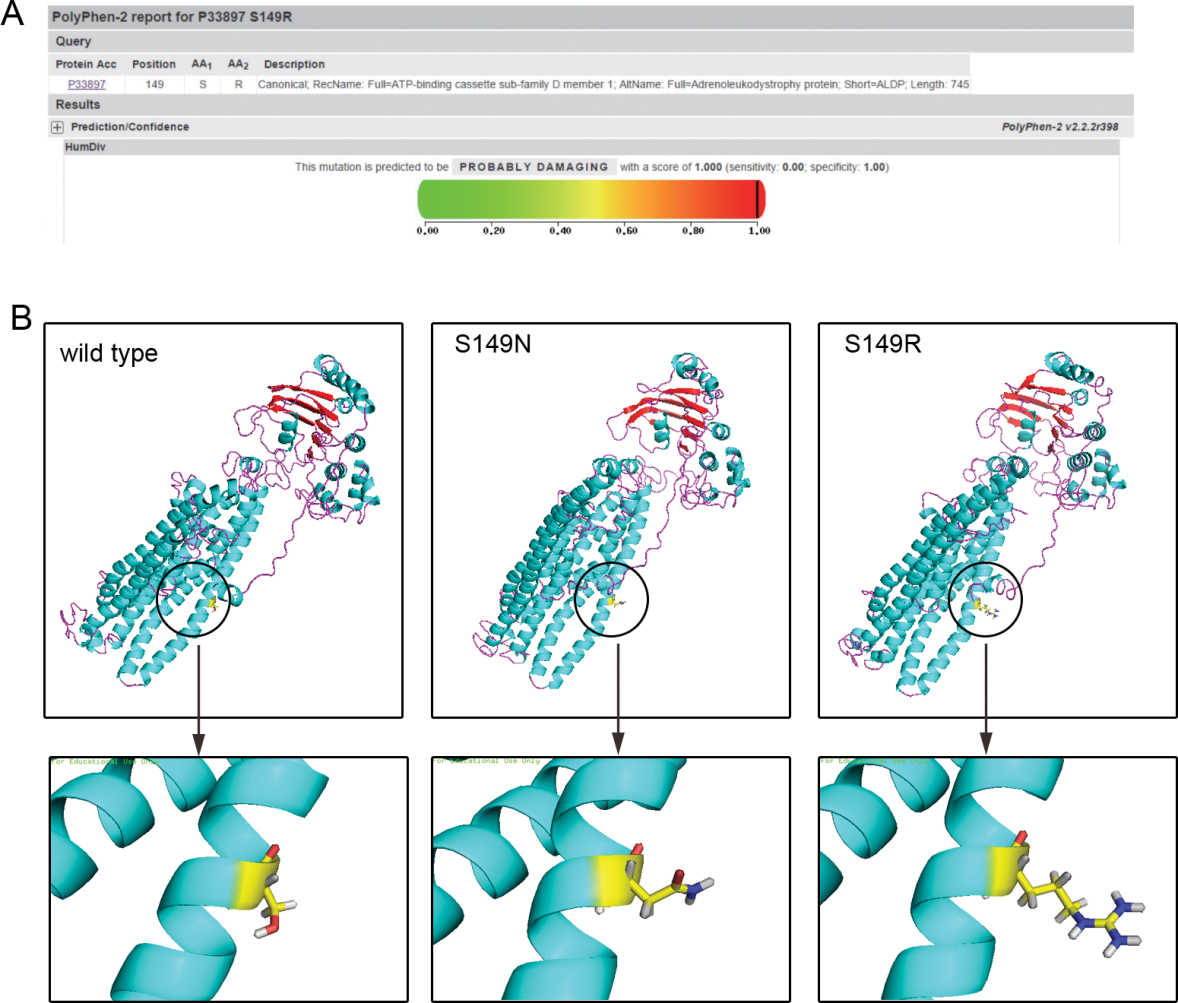
Bioinformatics analysis **(A)** The prediction of the mutation effect by the PolyPhen software program showing a damaging effect of the p.S149R mutation (score: 1.000 with a prediction: probably damaging). **(B)** 3D-structure of ALDP: left: wild-type ALDP; middle: change of the ALDP structure with novel missense mutations: p.S149N in a previous study; right: p.S149R in the present study.

According to sequencing alignment, the mutated amino acid is well-conserved among species. Compared to other ALDP proteins (Table 2), except for ABCD3 (PMP70), encoded by the *ABCD3* gene, the corresponding amino acid is N. According to PolyPhen-2, both S149R and S149N were strongly predicted to be pathogenic, with a score of 1.00. However, concerning the results of SIFT, S149N is predicted to be tolerated with a score of 0.37, as amino acids with probabilities <.05 are predicted to be deleterious. S149R is predicted to affect protein function with a score of 0.03 (Figure 3A).

**Table 2:** Protein alignment

ABCD1(Homo sapiens)	Leu	Pro	Ala	Thr	Phe	Val	Asn	Ser	Ala	Ile	Arg	Tyr	Leu	Glu	Gly
ABCD1(Pan troglodytes)	.	.	.	.	.	.	.	.	.	.	.	.	.	.	.
ABCD1(Mus musculus)	.	.	.	.	.	Ile	.	.	.	.	.	.	.	.	.
ABCD1(Rattusnorvegicus)	.	.	.	.	.	Ile	.	.	.	.	.	.	.	.	.
ABCD1(Oryctolaguscuniculus)	.	.	.	.	.	Ile	.	.	.	.	.	.	.	.	.
ABCD1(Susscrofadomesticus)	.	.	.	.	.	.	.	.	.	.	.	.	.	.	.
ABCD1(Macacamulatta)	.	.	.	.	.	.	.	.	.	.	.	.	.	.	.
ABCD1(Bos Taurus)	.	.	.	.	.	Ile	.	.	.	.	.	.	.	.	.
ABCD1(Caviaporcellus)	.	.	.	X	.	.	.	.	.	.	.	.	.	.	.
ABCD1(Feliscatus)	.	.	.	.	.	Ile	.	.	.	.	.	.	.	.	.
ABCD1(Canis lupus familiaris)	.	.	.	.	.	Ile	.	.	.	.	.	.	.	.	.
ABCD1(Equuscaballus)	.	.	.	.	.	Ile	.	.	.	.	.	.	.	.	.
ABCD1(Anoliscarolinensis)	.	.	.	.	.	.	.	.	.	.	.	.	.	.	.
ABCD2(Homo sapiens)	Ile	.	.	.	.	.	.	.	.	.	.	.	.	.	Cys
ABCD3(Homo sapiens)	Pro	Leu	Ile	Ser	Leu	.	.	Asn	Phe	Leu	Lys	.	Gly	Leu	Asn
ABCD4(Homo sapiens)	.	Asn	Ser	.	**-**	Leu	Lys	.	Phe	Asp	Gln	Phe	Thr	Cys	Asn
ABCD1(The patient)	.	.	.	.	.	.	.	Arg	.	.	.	.	.	.	.

Multiple protein alignment of the amino acid partial sequence from ALDP showing the highly conserved serine amino acid at position 149 (The position is marked in red).

As seen in Figure 3B, the three amino acids presented different properties, and the substitution caused a structural change in the protein. The mutation S149R made the 3D-structure of ALDP looser and detached. Protein with the mutation of S149N was much similar to the normal one, except for the branching in the lower right corner.

### Subcellular localization of the S149R mutant ALDP *in vitro*

**Figure 4:**
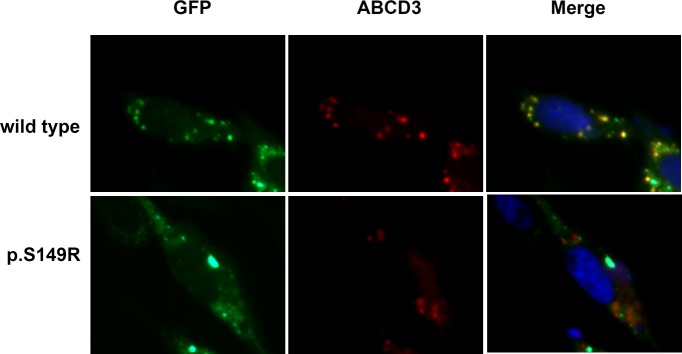
Subcellular localization of ABCD1 in CHO cells CHO cells were transfected with empty vector, pEGFP/ABCD1 (wild-type), or pEGFP/mutant-ABCD1 (p.S149R), and protein localization was observed by fluorescence microscopy. The ABCD3 proteins localized in the peroxisomes were detected using an anti-human ABCD3 antibody followed by secondary antibodies conjugated with Alexa Fluor 555 (red). The expressed ABCD1 proteins were detected as green fluorescent dots. The wild-type ABCD1 was localized to peroxisomes, but the mutant ABCD1 was diffuse in the cytosol. Original magnification: 600×.

The subcellular localization of proteins is strictly controlled in cells. ALDP is targeted to peroxisomes for the transport of VLCFA or their CoA derivatives [17]. To investigate if the mutation may influence the subcellular localization of ALDP, we analyzed the expression of the protein in CHO cells by immunofluorescence analysis. Peroxisomes were stained with an anti-ABCD3 antibody that recognized the ABCD3 protein, a main peroxisomal membrane protein. As shown in Figure 4, all cells exhibited ABCD3-positive immunofluorescent dots (red), suggesting the presence of intact peroxisomes in these cells. The distribution of wild-type ALDP (green) was completely superimposable on that of ABCD3 in the same cells, showing a yellow-brown color corresponding to a match between the green and the red punctate patterns, indicating that the wild-type ALDP was expressed and transported correctly to peroxisomes for the β-oxidation of VLCFA in CHO cells. In contrast, overexpressed S149R mutant ALDP showed a partially clustered pattern throughout the cytoplasm, as shown by a high density of green foci, indicating that the location of mutant ALDP was not peroxisomal and appeared to be mislocated to other organelles. These results demonstrated that the S149R mutation altered the targeting of ALDP to the peroxisome, which may have resulted in a reduction of peroxisomal fatty acid β-oxidation and finally an X-ALD phenotype.

## DISCUSSION

In this study, we identified a novel mutation S149R in the *ABCD1* gene associated with X-ALD. Interestingly, the affected patient presented an olivopontocerebellar form, the first case on the Chinese mainland. We also predicted the deleterious property of the mutation and concluded that the mutation could destroy the structure and function of ALDP, leading to defects in the degradation of VLCFAs in peroxisomes. Our findings provided further insights into the function of the *ABCD1*gene. The confirmation of an ALD diagnosis by genetic analysis is important to correlate genotype with phenotype. Moreover, these results will expand the spectrum of mutations and will provide more information for prenatal diagnosis and better patient counseling.

X-ALD can be classified into several types according to manifestations. Olivopontocerebellar ALD is the rarest form that could display similar features (e.g., onset age, pyramidal symptoms and sensory failure) as the AMN form, but most cases can be distinguished by cerebellar dominant symptoms [18]. Recently, Ogaki summarized all 34 cases of this form worldwide and found that approximately 88% of patients were from Japan and Korea [19]. However, only one case was identified in Taiwan, China [20]. Among the 34 cases of olivopontocerebellar ALD, only eight mutations have been identified, mainly distributed in exons 1,2,7 and 8 [20–22]. From the clinical symptoms of the proband (such as ataxia, dysarthria, and dysphagia), we suspected that he had olivopontocerebellar ALD, which was confirmed by genetic analysis. To our knowledge, this is the first case on the Chinese mainland, and a detailed molecular analysis might provide useful insights into the genotype-phenotype correlation.

Subsequently, we identified a novel mutation, S149R, in the *ABCD1* gene and predicted its disastrous effect on the ALDP protein. According to sequence alignment, the position (S149) is quite conserved among multiple species and ABCD proteins. According to properties of residues, arginine is basic and larger. However, serine is uncharged, polar and smaller. The differences in amino acid properties caused structural changes and probably altered ALDP function (Figure 3B). Moreover, we did not find this mutation in 100 controls and various databases. Gloeckner argued that an internal N-terminal sequence between amino acids 67 and 186 was critical in mediating the PEX19p interaction, which was involved in targeting ALDP to the peroxisomal membrane [23, 24]. The mutation S149R is within this region and likely causes the mistargeting of ALDP. Taken together, it appears that the variant is probably damaging instead of merely a polymorphism.

To further elucidate the effect of mutant S149R on the subcellular localization of ALDP, we used CHO cells as the host to express either wild-type or mutant ALDP and examined them with immunofluorescence microscopy. As endogenous ALDP in CHO cells cannot be detected under the microscope and the GFP tag is convenient for screening, we transfected wild-type or mutant pEGFP/ALDP in CHO cells. The wild-type ALDP-GFP exhibited a punctate staining pattern that was superimposable on the distribution of peroxisomes stained by marker ABCD3, suggesting the normal expression and distribution of ALDP-GFP. However, under the same conditions, the distribution of S149R ALDP-GFP did not overlap with that of ABCD3 in the same cells. It is likely that the abnormal pattern of mutant ALDP was the result of mislocalization to other places due to amino acid substitution.

To our knowledge, the S149R mutation has not been reported to date. Coincidently, several studies found another mutation, S149N, in the same position [4, 25, 26]. However, these previous studies did not present detailed clinical and bioinformatics materials of the patient, and the ALDP expression in cells of patients with the S149N mutation reduced only 23% compared with control cells [26]. Moreover, the prediction of the effect of the S149N mutation by PolyPhen and SIFT software programs showed an inconsistent result (SIFT score: 0.37 with a prediction: tolerated and PolyPhen score: 1.000 with a prediction: probably damaging). Therefore, the pathogenesis of this substitution requires further evidence, such as studies *in vivo*. We elaborately reported the patient in the present study to help further understand the function of the *ABCD1* gene.

A previous study reported that the mutations are not distributed equally in the *ABCD1* gene; in fact, most mutations accumulate in the transmembrane regions and the ATP-binding region [4]. The eight revealed mutations of the olivopontocerebellar form are located in those “hot spots”. This naturally occurring missense mutation (S149R) is located in exon 1 and is within TMD2. From previous reports on missense mutations in TMD2, we found that the phenotype varied, including CCALD, ACALD, AMN, AO and asymptomatic, and one patient was diagnosed by amniocentesis [4, 25, 27–32]. Several recent studies showed that the substrate-binding site of ABC proteins is located in the TMD [33] and that changing the charge or polarity of the amino acid residue could alter the specificity of the transporter. As S149R is located in the TMD, the substitution of this amino acid seems to affect substrate binding or transportation through ALDP.

Generally, X-ALD is clinically and genetically heterogeneous [12]. Large deletions, nonsense mutations or frame shift mutations, which result in the complete absence of a functional ALDP are found in patients covering the full spectrum of X-ALD phenotypes including very mild forms of late onset AMN [34]. More interestingly, six male patients of a family with a missense mutation, p.Pro484Arg presented five different phenotypes [35]. Thus, the total lack of a functional ALDP alone does not necessarily lead to the severe form of XALD. Environmental factors, such as head trauma may be required to initiate the pathologic process. Genetic factors are also involved in the clinical manifestation of X-ALD.

The main limitation of this study is the absence of screening for mutations in the other members of this family to determine whether this mutation is X-linked or *de novo*. It is estimated that *de novo* mutations may be more common than previously reported [36]. Confirming the type of mutation will benefit genetic counseling. In addition, a transfection study *in vivo* is not available in the present study, which is useful in the further study of the disease mechanism.

In conclusion, in this study, we reported the case of a Chinese man with olivopontocerebellar X-ALD, confirmed a novel mutation (c.447T>A; p.S149R), predicted its damaging effect and examined the subcellular localization of the mutant protein, which may expand the mutation spectrum, promote further understanding of the function of ALDP, and improve the quality of prenatal diagnosis and early prevention.

## MATERIALS AND METHODS

The research protocol was approved by the institutional review board of Shandong Provincial Hospital Affiliated to Shandong University, and written informed consent was obtained from the patient. All methods were performed in accordance with the relevant guidelines and regulations.

### DNA extraction and amplification

Genomic DNA was extracted from peripheral blood leucocytes of the patient using genomic DNA kit (QIAamp Blood DNA Mini Kit, QIAGEN, USA). Previously published primer sets were used to amplify the whole *ABCD1* gene, including all exons and its respective flanking regions [6]. Polymerase chain reaction (PCR) was performed in a 50-μl system including 4 μl dNTP, 5 μl 10^*^ PCR buffer, 0.3 μl Taq Hot Start (Takara Bio, Ohtsu, Japan), 8 μl genomic DNA and 1 μl 10 μM forward and reverse primers. The reaction condition contained an initial denaturation step at 94°C for 5 min subsequently followed by 40 cycles with denaturation at 94°C for 30 s, annealing at 60°C for 30 s and elongation at 72°C for 30 s. All amplifications were performed in an ABI9700 PCR amplifier (Life Technology, USA).

### Mutation analysis of *ABCD1* gene

The complete coding region of the *ABCD1* gene, including the intron-exon boundaries, was directly sequenced using a dye terminator cycle-sequencing system on an ABI 3500 DNA sequencer (Life technology, USA). The resulting sequences were compared to the corresponding wild-type sequences of *ABCD1* using AutoAssembler software (version 20; Perkin Elmer, Foster City, CA, USA). The +1 numbering of the *ABCD1* genomic DNA corresponds to the A of the ATG translation initiation codon. The mutations were designated using the recommendations of the Nomenclature Working Group, in which the genomic and cDNA sequence positions are designated by the prefixes g. and c., respectively. When sequence variants were detected, the exon was amplified from the genomic DNA extracted from 100 unrelated healthy individuals to determine whether the base variant was a polymorphic site.

### Bioinformatics analysis

To analyze the conservatism of *ABCD1* sequence, the multiple alignments of the ALDP peptide sequences were performed using the ClustalW (version 2.0.10) program, and the sequences of the different species as well as other ALDP proteins were obtained from the NCBI database. The protein functional effects of the novel missense mutation were predicted using PolyPhen-2 (http://genetics.bwh.harvard.edu/pph/) [37] and SIFT (http://sift.bii.a-star.edu.sg/) software [38]. The structure prediction was performed by Swiss Model(https://swissmodel.expasy.org/).

### Construction of mutant cDNA

ALDP-GFP expression vector (pEGFP/ALDP) was kindly provided by Dr. Imanaka, University of Toyama, Toyama, Japan [39]. A mutant version of *ABCD1*containing a missense mutation (pEGFP/mutant-ALDP) was prepared with a Quickchange^TM^ site-directed mutagenesis kit (Stratagene, La Jolla, CA, USA) using pEGFP/ALDP as a template. The primers used were designed based on their sequences (available upon request). The mutation in the constructions was confirmed by DNA sequencing on an ABI PRISM 310 DNA sequencer (Perkin Elmer Life Science, Wellesley, MA).

### Cell culture and transfection

Transfection of pEGFP/ALDP and pEGFP/mutant-ALDP was carried out to obtain cells expressing wild-type ALDP-GFP and mutant ALDP-GFP, respectively. CHO cells (5^*^10^5 cells) were cultured in Ham's F-12 medium supplemented with 10% FBS, 70 μg/mL of penicillin, and 140 μg/mL of streptomycin and transfected with 5 μg of pEGFP/ALDP or pEGFP/mutant-ALDP. Transfection assays were performed using Lipofectamine 2000 reagent (Invitrogen, Carlsbad, CA).

### Immunofluorescence study

CHO cells attached to coverslips were washed with PBS and fixed in 4% paraformaldehyde for 10 minutes. Cells were permeabilized with 0.1% Triton™ X-100 for 10 minutes and blocked with 10% normal donkey serum for 30 minutes, incubated with the primary antibody (mouse anti-human ABCD3, 1: 250 dilution, Santa Cruz) overnight at 4°C and then incubated for 1 h at room temperature with Alexa Fluor® 555-conjugated donkey anti-mouse IgG (1:500 dilution, Thermo Fisher Scientific). The staining signals for PMP70/ABCD3 (70-kDa peroxisomal membrane protein) were specific, as incubation with nonimmune IgG showed no detectable fluorescence under similar conditions (data not shown). The nucleus was stained with DAPI (4′,6-diamidino-2-phenylindole). Specimens were imaged with a fluorescence microscope (Axiovert 100M Zeiss). The images were captured at 600X magnification.

## Abbreviations

X-ALD: X-linked adrenoleukodystrophy
ABCD1: ATP-binding cassette protein subfamily D1
AMN: adrenomyeloneuropathy
AO: Addison-only
ALDP: adrenoleukodystrophy protein
VLCFA: transporting very long chain fatty acid
MRI: magnetic resonance imaging
CT: computed tomography
PCR: polymerase chain reaction
DAPI: 4′,6-diamidino-2-phenylindole
